# Preparation of chaperone-loaded neural stem cell-derived extracellular vesicles to reduce protein aggregation in Huntington’s disease cellular models

**DOI:** 10.1016/j.xpro.2023.102134

**Published:** 2023-02-25

**Authors:** Bhagyashree S. Joshi, Inge S. Zuhorn

**Affiliations:** 1 Department of Biomedical Engineering, University of Groningen, University Medical Center Groningen, A. Deusinglaan 1, 9713 AV Groningen, the Netherlands

**Keywords:** Cell Biology, Cell Separation/Fractionation, Health Sciences, Microscopy, Molecular Biology, Gene Expression, Neuroscience, Molecular/Chemical Probes, Protein Biochemistry, Stem Cells

## Abstract

Here, we present a protocol using genetic engineering techniques to prepare small extracellular vesicles (sEVs) enriched in the chaperone protein DNAJB6. We describe steps to prepare cell lines overexpressing DNAJB6, followed by the isolation and characterization of sEVs from cell conditioned media. Further, we describe assays to examine effects of DNAJB6-loaded sEVs on protein aggregation in Huntington’s disease cellular models. The protocol can be readily repurposed to study protein aggregation in other neurodegenerative disorders or extended to other therapeutic proteins.

For complete details on the use and execution of this protocol, please refer to Joshi et al. (2021).^1^

## Highlights

• Cost-effective protocol for loading chaperone proteins into extracellular vesicles (EVs)
• Preparation of cell lines overexpressing therapeutic protein cargo and isolation of EVs
• Assays to study anti-aggregation effects of EVs in Huntington’s disease cell models
• Applicable to assess functional effects of EVs in other protein aggregation diseases

## Abstract (editor highlights)

Publisher’s note: Undertaking any experimental protocol requires adherence to local institutional guidelines for laboratory safety and ethics.

## Graphical abstract

**Figure:**
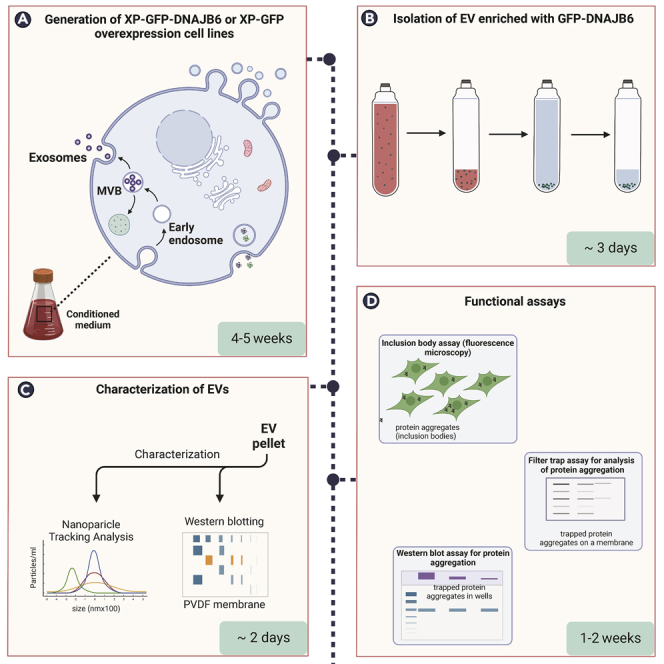

## Before you begin

This protocol describes methods for isolating small extracellular vesicles (sEVs) from neural stem cell line C17.2 overexpressing the chaperone protein DNAJB6b and studying the effect of these sEVs on protein aggregation in *in vitro* cellular models of Huntington’s disease.

### Preparation of plasmids expressing DNAJB6

**Timing: ∼ 4 days**

This section describes how to prepare plasmids expressing XPackTM-tagged DNAJB6.

1. Purchase XPack™ CMV-XP-MCS-EF1-Puro Cloning Lentivector from SBI biosciences (XPAK510PA-1). For more details about the vector, please refer to the company manual (https://www.systembio.com/wp/wp-content/uploads/2020/10/Manual_XPack_WEB-1.pdf).
2. Amplify GFP and GFP-DNAJB6 fragments by PCR using pcDNA5/FRT/TO GFP-DNAJB6b (Addgene#19501). Follow the recipe below for PCR-

**Table:** PCR reaction master mix

Reagent	Amount (μL)
DNA template (100 ng)	1
Herculase II fusion DNA polymerase	0.5
Primer 1 (see key resources table) (10 μM)	1.25
Primer 2 or 3 (see key resources table) (10 μM)	1.25
5× Herculase II reaction Buffer	10
dNTP mix (25 mM each dNTP)	0.5
ddH_2_O	Add up to 50
Total volume	50

**Table:** PCR cycling conditions

Steps	Temperature	Time	Cycles
Initial Denaturation	95	2 min	1
Denaturation	95°C	10 s	25–35 cycles
Annealing	60°C	20 s	
Extension	72°C	30 s per kb	
Final extension	72°C	3 min	1
Hold	4°C	forever

3. Digest the respective PCR segments and the parent lentiviral vector with XhoI-HF and EcoRI-HF. The PCR fragments are 733 bp (GFP) and 1,471 bp (GFP-DNAJB6) in size. Prepare the digestion reaction mixture as shown below and incubate at 37°C for 2 h.

**Table:** Restriction digestion reaction mix

Reagent	Amount (μL)
CutSmart Buffer (10×)	5
DNA template	10 μg equivalent
XhoI	1.5
EcoRI	1.5
ddH_2_O	Add up to 50
Total volume	50

4. Purify the digested PCR products using QIAquick PCR & Gel Cleanup Kit (https://www.qiagen.com/br/resources/download.aspx?id=a72e2c07-7816-436f-b920-98a0ede5159a&lang=en).
5. Run the digested vector on an agarose gel at 200 V until resolved properly to separate the DNA fragment generated by XhoI and EcoRI restriction from the vector. There should be two bands on the gel of differing sizes.
6. Under UV illumination, excise out the bigger band, which represents the remaining vector DNA to be inserted with the PCR amplicons. Using QIAquick PCR & Gel Cleanup Kit (https://www.qiagen.com/br/resources/download.aspx?id=a72e2c07-7816-436f-b920-98a0ede5159a&lang=en), elute the DNA from the gel piece.
7. Insert the GFP and GFP-DNAJB6 PCR fragments into the cut vector using ligation to produce pCMV-XP-GFP-EF1-Puro and pCMV-XP-GFP-DNAJB6-EF1-Puro, respectively. Set up the ligation reaction mixture given below and incubate at RT for 10 min.

**Table:** Ligation reaction mix

Reagent	Amount (μL)
NEB Ligation Buffer (10×)	5
NEB Ligase	10 μg equivalent
Cut vector (100 ng)	1.5
PCR (50 ng)	1.5
ddH_2_O	Add up to 50
Total volume	50

8. Transform the ligation mixture into bacteria (DH5α) and plate on agar plates containing ampicillin, incubate for 18 h at 37°C.
9. The next day, screen 10 colonies with a colony PCR using Green GoTaq PCR reaction (Promega)

**Table:** PCR reaction master mix

Reagent	Amount (μL)
5× GoTaq Green reaction buffer	2
Primer 1 (see key resources table) (10 μM)	0.1
Primer 2 or 3 (see key resources table) (10 μM)	0.1
GoTag DNA polymerase	0.1
dNTP mix (10 mM each dNTP)	0.2
ddH_2_O	Add up to 10
Colony	Scrape with a tip and add to the PCR tube
Total volume	10

**Table:** PCR cycling conditions

Steps	Temperature	Time	Cycles
Initial Denaturation	95	2 min	1
Denaturation	95°C	30 s	25–35 cycles
Annealing	60°C	30 s	
Extension	72°C	1 min per kb	
Final extension	72°C	5 min	1
Hold	4°C	Forever

**Figure 1:**
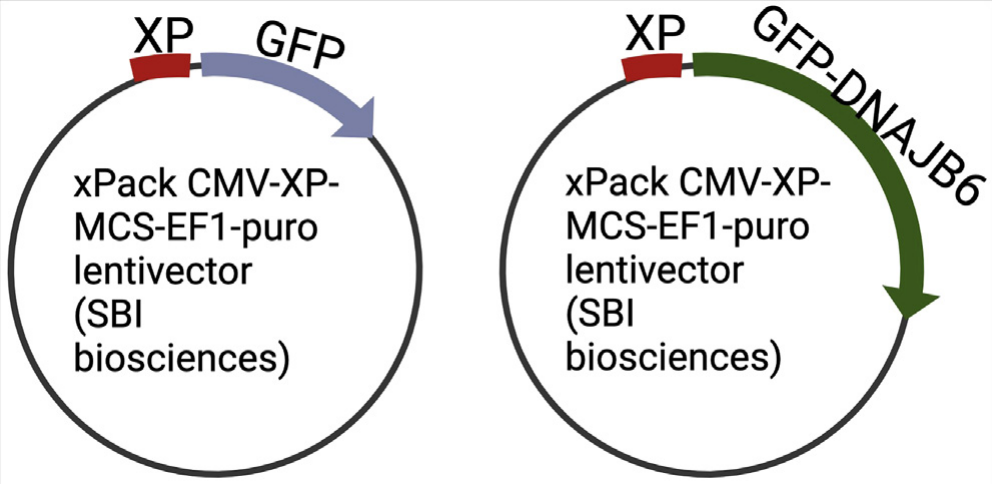
Plasmids used in this study

10. Select three clones that give a clean PCR band at expected size.
11. Inoculate the three clones separately in 3 mL LB medium containing ampicillin and grow overnight at 37°C.
12. Isolate plasmids using Promega plasmid miniprep kit (https://nld.promega.com/resources/protocols/technical-bulletins/101/pureyield-plasmid-miniprep-system-protocol/).
13. Send clones for DNA sequencing and select one that shows no mutations in the insert sequence.
14. The final vectors are shown in Figure 1.

### Exosome-depleted complete medium preparation

**Timing: ∼ 18 h**

This section describes how to deplete exosomes from complete medium for the preparation of exosome collection media.

15. Prepare DMEM with 10% fetal bovine serum (FBS) with 1% penicillin-streptomycin.
16. Carefully disinfect all the ultracentrifuge (UC) tubes (38.5 mL, Certified Free Open-Top Thinwall Polypropylene Tube, 25 × 89 mm - 50Pk, C14285) by spraying ample amounts of 70% ethanol on both the inner and outer sides.
17. Likewise, spray the Beckman SW32i rotor tubes with 70% ethanol.
18. From now onwards, work under the laminar flow. Keep the tubes and holders sprayed with ethanol under the hood and let them dry and disinfect.
19. Add media to all the UC tubes until 2 cm from the edge (to avoid spoilage).
20. Tighten the caps properly and carefully place the tubes in the holders.
21. Balance the tubes with the caps closed, and re-balance, if necessary, by adding or removing media under the laminar flow.
22. Carefully place the balanced holders in the same rotor in the UC and Sorvall Discovery 90SE, centrifuge at 110,000 g for 16 h at 4°C to spin down EVs present in the FBS.
23. Collect the resulting supernatant by pipetting and filter sterilize through a 0.2 μm filter.
24. Store the resulting supernatant at 4°C for a maximum of 2 weeks.

***Note:*** Alternatively, 100% FBS can be ultracentrifuged at 110,000 g for 16 h at 4°C, and the supernatant can be stored at -80°C for long term use. Although this procedure is more time-effective, the viscosity of pure FBS may cause the elimination of various proteins and other FBS components that can pellet as aggregates. This may affect the cell viability and activity.^2^

### Cell culture conditions

25. Prepare DMEM with 10% FBS, 5% Horse serum, and 1% Penicillin streptomycin. Make sure that the components are mixed properly.
26. Store this complete medium at 4°C for max. 2 months or until the media has turned red (which means it is too alkaline to use).
27. Subculture C17.2 cells in complete medium and incubate overnight until all cells are attached to the surface of the culture flask.

**CRITICAL:** Generally, for extracellular vesicle isolation, cells are seeded in EV-depleted medium (Section Isolation of small extracellular vesicles from cell culture). However, C17.2 cells are sensitive to serum deprivation when cultured in EV-depleted medium and tend to differentiate. Hence, it is advisable to seed cells in complete medium. The next day once cells have adhered to the surface, rinse cells thoroughly with Hank's Balanced Salt Solution (HBSS) (two rinses) and then add EV-depleted medium. Isolate EVs after 2 days of incubation.

After overnight incubation the cells should be ∼60% confluent. Wash twice with HBSS and proceed to adding the EV-depleted medium. For T162 flask, add 20 mL medium (which will just cover the surface) to concentrate the sEV sample.

**CRITICAL:** C17.2 neural stem cells multiply very fast. When crowded, they tend to differentiate into elongated neuronal cells. It is important to control the cell density while culturing them for regular passaging as well as extracellular vesicles collection to prevent the presence of differentiated cells. 60% cell confluency is a good moment for the addition of EV-depleted medium, and 2 days incubation time is sufficient to get a good amount of EVs from 10 T162 flasks (∼2 μg/μL, ∼50 μL total volume).

28. Culture HEK293T cells with DMEM, 10% FBS and 1% Pen-strep. Passage the cells every 3 days or when 90% confluent.

## Key resources table

**Table:** 

REAGENT or RESOURCE	SOURCE	IDENTIFIER
**Antibodies**		

Rat monoclonal anti-Lamp2b ABL-93 (1:1000)	Developmental Studies Hybridoma Bank	AB-2134767
Rabbit monoclonal anti-CD9 (1:200)	Abcam	ab92726
Rabbit monoclonal anti-calnexin (1:1000)	StressGen	ADI-SPA-860
Mouse monoclonal anti-GFP (1:2000)	Clontech	632381
Rabbit polyclonal anti-DNAJB6 (1:1000)	Custom made	NA
IRDye® 680RD Goat anti-Mouse IgG Secondary Antibody (1:5000)	Odyssey Li-COR	LI 926-68070
IRDye® 800CW Goat anti-Rabbit IgG Secondary Antibody (1:5000)	Odyssey Li-COR	LI 926-32211
IRDye® 800CW Goat anti-Rat IgG Secondary Antibody (1:5000)	Odyssey Li-COR	LI 926-32219
Goat anti-Rabbit IgG (H+L) Cross-Adsorbed Secondary Anitbody, Alexa Fluor 546 (1:500)	Invitrogen	A-11010

**Chemicals, peptides, and recombinant proteins**		

Doxycycline	Sigma	D9891
DMEM high glucose	Gibco™	11965092
FBS	Gibco™	16000044
Horse serum, heat inactivated, New Zealand origin	Gibco™	26050070
Penicillin-Streptomycin-Glutamine (100×)	Gibco™	10378016
Puromycin	Invivogen	ant-pr-5
HBSS, no calcium, no magnesium, no phenol red	Gibco™	14175095
Trypsin-EDTA (0.25%), phenol red	Gibco™	25200056
Bovine serum albumin	Sigma	A7906
DL-dithiothreitol (DTT)	MedChemExpress	HY-15917
Dulbecco’s phosphate-buffered saline	Gibco™	14190144
Phosphate buffered saline	Gibco™	10010002
Tween-20	Sigma	P1379
Sodium dodecyl sulfate (SDS)	Sigma	L3771
2× Laemmli sample buffer	Biorad	1610737
protease inhibitors	Roche	11697498001

**Critical commercial assays**		

SG cell line 4D nucleofector kit	Lonza	V4XC-3032
Bio-rad DC protein assay kit	Bio-Rad	5000114
ECL	GE Healthcare	RPN2232
QIAquick PCR & Gel Cleanup Kit (100)	Qiagen	28506
PureYield™ Plasmid Miniprep System	Promega	A1222

**Experimental models: Cell lines**		

Human HEK293T (passage number <45)	Sigma-Aldrich	12022001
Mouse C17.2 neural stem cells (passage number <15)	Sigma-Aldrich	07062902
Mouse C17.2-XPack-GFP(passage number <15)	This paper	N/A
Mouse C17.2-XPack-GFP-DNAJB6b (passage number <15)	This paper	N/A

**Oligonucleotides**		

Primer 1: XP-GFP/XP-GFP-DNAJB6 Forward	This paper	GCGCTCGAGCATGGTGAGCAAGGGCGAGGA
Primer 2: XP-GFP Reverse	This paper	GCGGAATTCTTACTTGTTATCCAAGCGCAGCAGC
Primer 3: XP-GFP-DNAJB6 Reverse	This paper	GCGGAATTCTTACTTGTACAGCTCGTCCATGCC

**Recombinant DNA**		

XPack CMV-XP-MCS-EF1-Puro	SBI Biosciences	XPAK510PA-1
pcDNA5/FRT/TO GFP-DNAJB6b	Hageman et al.^3^	N/A
pCMV-XP-GFP-EF1-Puro	This paper	N/A
pCMV-XP-GFP-DNAJB6-EF1-Puro	This paper	N/A
pEGFP-Q74	Narain et al.^4^	Addgene plasmid #40262

**Software and algorithms**		

Nanoparticle Tracking Analysis (NTA) software 3.0	Malvern, Worcestershire, United Kingdom	https://www.malvernpanalytical.com/en/products/technology/light-scattering/nanoparticle-tracking-analysis
ImageJ	Schneider et al.^5^	https://imagej.nih.gov/ij/download.html
GraphPad Software Prism 5	GraphPad software, Inc.	https://www.graphpad.com/scientific-software/prism/

**Other**		

38.5 mL, certified free open-top thinwall polypropylene tube, 25 × 89 mm (ultracentrifuge tube)	Beckman Coulter Life Sciences	C14285
14 mL, certified free open-top thinwall polypropylene tube, 14 × 95 mm (ultracentrifuge tube)	Beckman Coulter Life Sciences	C14287
Allegra A-15R centrifuge	Beckman Coulter Life Sciences	C63126
Sorvall Discovery 90SE ultracentrifuge	Sorvall	45912-2
Fluorescence microscope	Leica DMI 6000B	https://www.leica-microsystems.com/products/light-microscopes/p/leica-dmi6000-b/
Confocal microscope	Leica SP8	https://www.leica-microsystems.com/products/confocal-microscopes/p/leica-tcs-sp8/
Nanosight LM14	Malvern, Worcestershire, United Kingdom	https://www.malvernpanalytical.com/en/products/product-range/nanosight-range/nanosight-ns300
Odyssey® Infrared Imaging System	Li-COR	https://www.licor.com/bio/support/contents/applications/western-blots/fluorescent-western-blot-detection-protocol.html?Highlight=on-cell western assay
Faramount Mounting Medium, Aqueous	Agilent Dako	S302580-2
Cellulose Acetate membrane filters, 0.2 Micron	Sterlitech	SKU CA023001
13 mm glass coverslips	VWR	631-0148
Odyssey blocking buffer	Li-COR	927-40000
JetPEI®	Polyplus	10110-N
Bio-Dot® SF Apparatus	Bio-Rad	1706542
Costar® 24-well Clear TC-treated Multiple Well Plates, Individually Wrapped, Sterile	Corning	3524
Costar™ 6-well Clear TC-treated Multiple Well Plates, Individually Wrapped, Sterile	Corning	3516
PVDF membrane	Millipore	IPFL00010

## Step-by-step method details

### Preparation of cell lines overexpressing therapeutic protein cargo

**Timing: ∼ 4 weeks**

**Figure 2:**
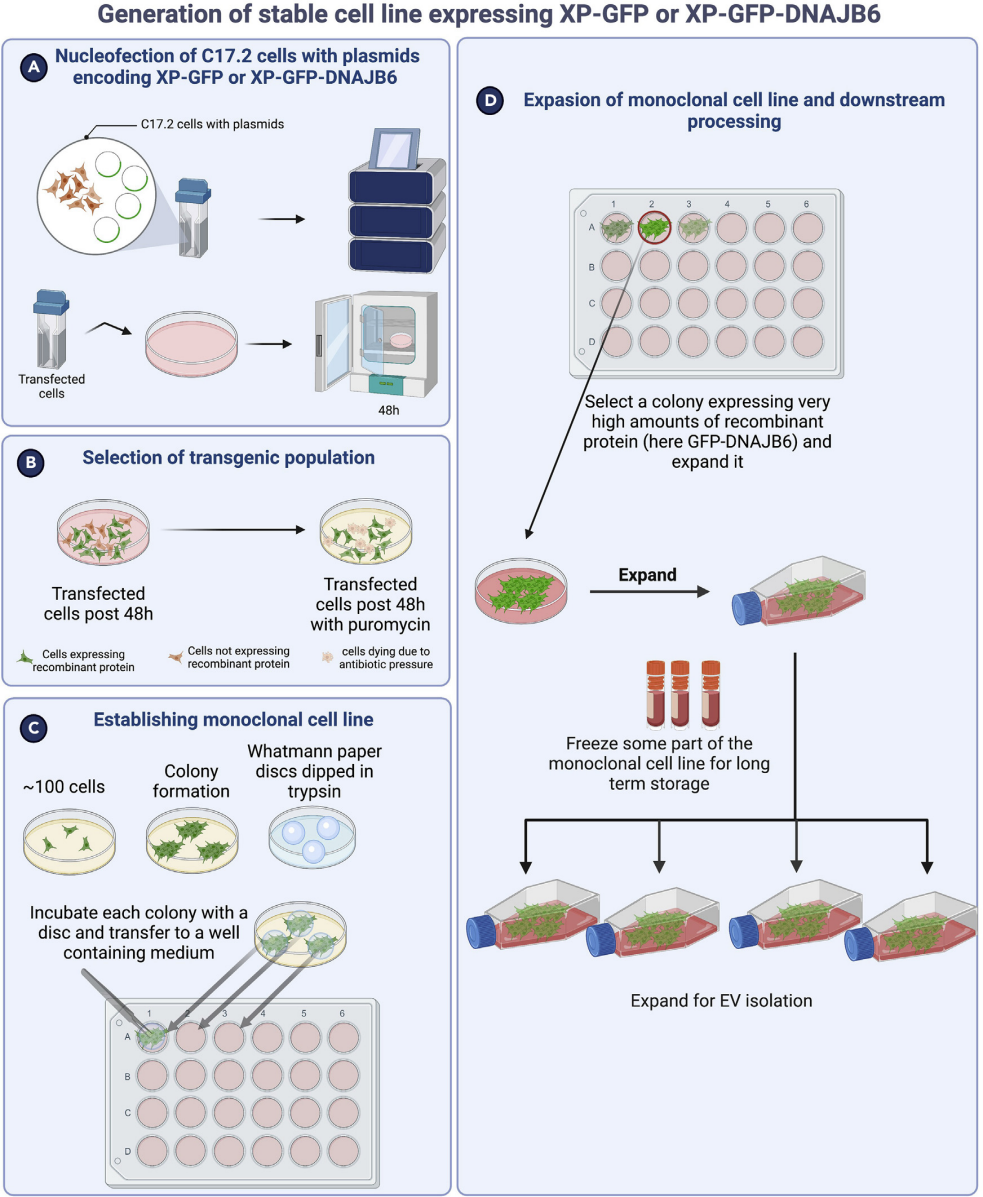
Schematic overview of generation of monoclonal stable cell line expressing the recombinant protein of interest (A) Nucleofection of plasmid encoding recombinant protein of interest into C17.2 cells using Nucleofection. (B) Selection of the transgenic population by applying antibiotic pressure. (C) Establishing a monoclonal stable cell line. (D) Selection of the clone expressing high amounts of recombinant protein of interest.

This process details the establishment of stable C17.2 cell lines overexpressing DNAJB6 at high amounts (Figure 2).

We present here a method of monoclonal cell preparation using a paper disc method, which is a labor- and cost-effective method of generating a monoclonal cell line without the need for sophisticated equipment such as a FACS sorter.

**CRITICAL:** In our hands, lipofection and lentiviral transduction did not result in stable C17.2 cell lines with high exogenous protein expression (which is necessary for high protein loading in EVs, which in turn would improve the therapeutic efficacy of EVs). Nucleofection gave the best results in terms of high protein expression. Hence, we have used nucleofection in this protocol.

***Note:*** Nucleofection, combining cell type-specific Nucleofector Solution and electrical pulses, facilitates DNA entry into the nucleus, increasing the chance of genomic integration upon antibiotic selection.

1. Seed 2–3 × 10^6^ C17.2 cells per 15 cm cell culture dish (1 for each plasmid nucleofection) and incubate at 37°C/ 5% CO_2_ overnight resulting in 3–4 × 10^6^ cells/ 80% confluency.
2. Remove the culture medium from the cells and wash cells once with Dulbecco’s phosphate-buffered saline (DPBS), using at least the same volume of PBS as culture medium.
3. Proceed with harvesting by incubating cells at 37°C with trypsin/EDTA for 3 min or until all cells are detached from the culture dish surface.
4. Add complete medium to the dish, and pipette up and down until you get a homogenous single-cell suspension.
5. Take 10 μL from the single-cell suspension and determine the cell density with a hemocytometer.
6. Centrifuge 1 × 10^6^ cells/condition at 200 g for 10 min at room temperature (RT). During this time, prepare nucleofection materials:
  a. Add Supplement solution to the Nucleofector™ Solution (supplied in the kit).
  b. Start 4D-Nucleofector™ System (Lonza) and upload DN-100 experimental parameter file as for C17.2 cells, SG transfection solution and Nucleofector™ program DN100 showed good results (refer to this protocol https://bioscience.lonza.com/lonza_bs/CH/en/download/product/asset/21623, however note that the program is DN100 instead of that mentioned in the protocol, follow the procedure for 100 μL Nucleocuvette™).
  c. Fill 10 mL complete culture medium in each 10 cm cell culture plate (one plate per condition) and pre-incubate/equilibrate plates in a humidified 37°C/5% CO2 incubator.
  d. Pre-warm an aliquot of culture medium to 37°C. Prepare plasmid DNA.
7. After 10 min of centrifugation, remove the supernatant and resuspend the cell pellet carefully in RT 4DNucleofector™ Solution.
8. Add 5 μg of plasmid DNA to this suspension (max. 10% of final sample volume) and transfer to a Nucleocuvette™ Vessel.
9. Repeat this procedure for the rest of Nucleocuvette™ Vessels as required.

***Note:*** As prolonged incubation of cells in Nucleofector™ Solution may lead to reduced transfection efficiency and viability it is important to work as quickly as possible. Avoid air bubbles while pipetting.

**CRITICAL:** Make sure to have <85% cell confluency when collecting cells for nucleofection. Cell confluency >85% will result in reduced transfection efficiencies.

***Note:*** C17.2 cells tend to grow faster at higher passage number, and eventually tend to differentiate into elongated cells that stop dividing. Hence, it is advisable to use cells with low passage number (<15) to generate a stable cell line by means of nucleofection.

10. Tap the Nucleocuvette™ Vessels gently to cover the sample at the bottom of the cuvette.
11. Close the lid and place the cuvette into the retainer of the 4D-Nucleofector™ X Unit in proper orientation.
12. Press *Start* on the display of the 4D-Nucleofector™ Core Unit (for details, please refer to the device manual https://bioscience.lonza.com/lonza_bs/NL/en/document/31856).
13. Remove the cuvette from the retainer after completing the run.
14. Resuspend the cells with pre-warmed medium and mix by pipetting gently only two to three times using the supplied pipettes with the kit avoiding bubble formation.
15. Add the cells to the cell culture plates ready from 6c.
16. Incubate the cells in humidified 37°C/5% CO2 incubator. Gene expression is often detectable after only 4–8 h.
17. Two days post-transfection, check the transfection efficiency.

***Note:*** In our hands, it was ∼50% with 40%–60% cell viability.

18. Replace the complete medium with the one supplemented with 3 μg/mL puromycin.

***Note:*** Generally, 1 μg/mL puromycin is sufficient for polyclonal cell line preparation. However, to select cells with high expression of recombinant protein, a higher puromycin concentration is recommended).

**Figure 3:**
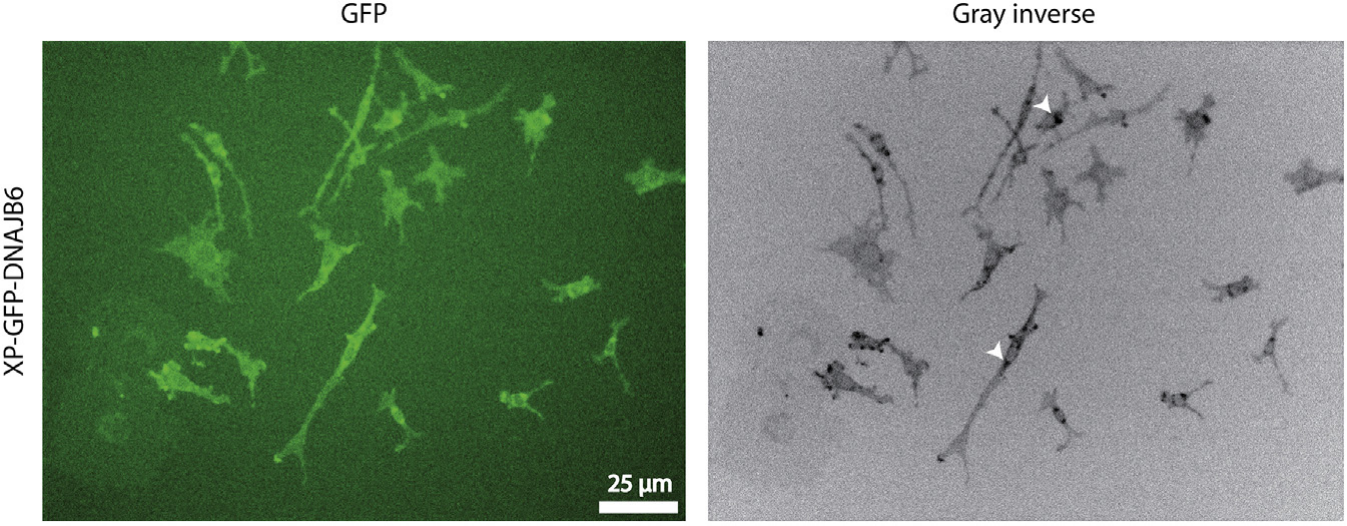
Polyclonal cells expressing XP-GFP-DNAJB6 protein post-nucleofection and post-antibiotic application GFP and Gray inverse images show the same field of view. White arrows highlight the perinuclear location of accumulated XP-GFP-DNAJB6 in multivesicular bodies where sEVs are generated.

***Note:*** These are polyclonal cells with various degrees of recombinant protein expression. The accumulated signal (white arrows in inverse grayscale image) indicates the localization of the recombinant protein in multivesicular bodies.

19. Over the next few days non-transfected cells will die. To remove dead cells and cell debris, refresh the medium with fresh medium with puromycin every other day.
20. After a couple of weeks, only cells that have incorporated the exogenous DNA into their genome will have survived the antibiotic selective pressure. A representative fluorescence micrograph of cells expressing XP-GFP-DNAJB6 is shown in Figure 3.

21. Wash cells with HBSS thoroughly to remove cellular debris, trypsinize cells, and divide over 3 dishes. Return cells to incubator for 2 days.
22. After 2 days incubation, trypsinize cells with 1.5 mL trypsin/EDTA, and resuspend cells in 10 mL complete medium.
23. Keep 2 mL cell suspension aside, and pellet down the 8 mL cell suspension at 200 g for 5 min. Discard the supernatant, add 4 mL freezing medium (complete medium with 10% DMSO) and divide over 4 cryogenic vials and freeze the cells.

***Note:*** These are backup polyclonal cell line vials, which can be thawed in case of contaminations during the monoclonal cell line establishment.

24. Count cells in the 2 mL cell suspension from (23), and seed 50 and 100 cells in 2 different 10 cm dishes. Add 20 mL complete medium. Place the dishes in the incubator.
25. 24 h post seeding, check if cells have attached to the bottom of the dish and if they look healthy. If everything seems okay, return the dish to the incubator and leave it untouched for 6 more days.
26. After 1 week post seeding, check for colonies. By now, there should be single colonies visible. They should grow apart from each other as this is important for picking single colonies later on.
27. There should be at least 100 cells per colony. Keep checking the size of the colonies every now and then. Generally, colonies will be ready for picking 2 weeks post-seeding.
28. In the meantime, cut out Whatman paper (number 1) discs with a punching machine and autoclave them.
29. At the day of colony picking, seal the dish with parafilm. Clean the microscope stage with 70% ethanol and screen the dish for colonies with GFP fluorescence. Encircle the colonies (> 12 clones) with highest fluorescence on the bottom of the dish using a marker pen.
30. Remove the parafilm and place the dish back in the incubator. Warm up cell culture medium, trypsin and HBSS. Take 5 mL trypsin in a small dish. Put 12–15 autoclaved paper discs in the lid of the dish.
31. Distribute 500 μL complete medium per well in a 24 well plate. Label the wells with numbers.
32. Take the dish with colonies out of the incubator and place it in the laminar flow. Wash with HBSS twice. Flame the tweezers twice and let them cool down.
33. Meanwhile, remove HBSS from the dish. From this time onwards, one has to work fast in order to prevent cells from drying. Take a paper disc, dip it in trypsin/EDTA and put on the encircled colony. Repeat this for all the colonies. Keep the dish in the incubator for 3 min.
34. Pick the paper discs one by one and put one disc per well in the 24 well plate.
35. After transfer of all discs to the 24 well plate, pipette up and down medium in every well with a separate tip with a 1 mL micropipette to rinse the discs with the medium.
36. Place the 24 well plate in the incubator.
37. Keep checking this plate daily. It will take 4–5 days before cells are detected in the wells. Once the wells are around 70% confluent, discard the Whatman paper discs, transfer cells from one well in 24 well plate to one well in 6 well plate.
38. Select clones that show the maximum amount of GFP and GFP-DNAJB6 expression at the expected localization of XPack™ tagged proteins, i.e., at the plasma membrane and endosomal structures.

***Note:*** These clones are your parental cell lines for XP-GFP and XP-GFP-DNAJB6 loaded extracellular vesicle production, respectively.

39. Transfer each of the selected clones to a separate 10 cm dish, and let cells grow until 80% confluence. Expand each 10 cm dish to 3 10 cm dishes. Freeze cells from 2 dishes in 5 cryogenic vials. Use the remaining 10 cm dish for downstream processing.

**CRITICAL:** Expression of XPack™ tagged GFP fusion proteins produces a cellular distribution pattern similar to sEV marker proteins, e.g., the tetraspanin CD9. Specifically, some of the GFP signal is localized on the plasma membrane and most is localized at multivesicular endosomes.^1^

### Cleaning coverslips for microscopy

**Timing: ∼ 30 min**

**Figure 4:**
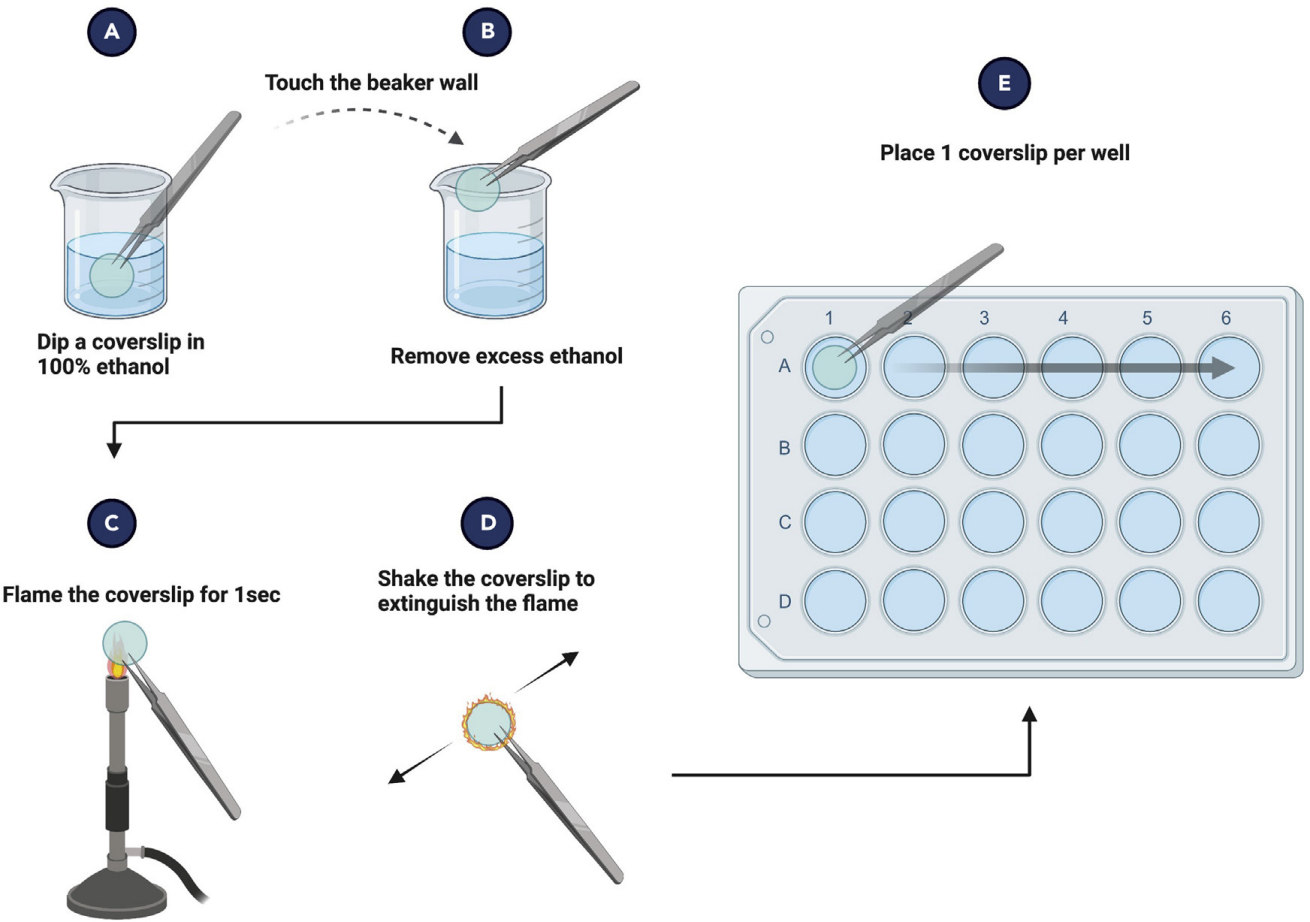
Schematic overview of the cleaning coverslips for microscopy (A) Dip a coverslip in 100% ethanol. (B) Remove excess ethanol by touching the coverslip to the beaker wall. (C) Flame the coverslip by holding it in the flame for a second. (D) Extinguish the flame by shaking the coverslip. (E) Place 1 coverslip per well.

This section describes the preparation of cleaning coverslips for microscopy (Figure 4).

We use 13 mm glass coverslips (VWR) to seed cells on in 24 well plates. For quick and effective cleaning, we advise the procedure below which involves dipping the coverslips in 100% ethanol and flaming them to remove dirt and for sterilization.

40. Use dissecting forceps to hold coverslips gently but firmly to avoid any breakage.
41. Aliquot 100 mL 100% ethanol in a Scott Duran bottle. Transfer 10–15 mL 100% ethanol from this into a small glass beaker under the cleaned laminar flow.
42. Place a 24 well plate under the laminar flow.
43. Take out one coverslip from its case with the forceps, and dip it in the ethanol, ensuring that it is fully submerged.
44. Remove the coverslip and touch the beaker wall to remove excess ethanol.
45. Start the flame under the hood and swiftly pass the coverslip through the flame for the residual ethanol on the coverslip to catch fire. Stop the flame.

***Note:*** Keep the beaker with ethanol away from the flame!

46. Keep moving the forceps slowly in the air until the flame is extinguished.

**CRITICAL:** It is important to keep moving the forceps in order to avoid the breakage of coverslips due to overheating. Keep your hand with forceps away from the beaker with ethanol to avoid fire mishaps. Alternatively, use 70% ethanol and UV light for sterilization.

47. Place one coverslip per well in the sterile 24-well plate.
48. The wells containing coverslips are now ready for cell seeding.

### Characterization of donor cell lines using immunostaining

**Timing: ∼ 3 days**

This section describes how to characterize the exosome donor cell lines for the presence of exosome marker proteins using immunostaining.

***Note:*** Characterization of donor cell lines using immunostaining for extracellular vesicle markers – It needs to be ensured that XP-tagged proteins are localized in multivesicular bodies and colocalize with extracellular vesicle marker proteins

49. Trypsinize wild type (WT), XP-GFP, and XP-GFP-DNAJB6b C17.2 cells, count and seed 3 × 10^4^ cells in each well with coverslip in the 24 well plate and incubate for 24 h at 37°C.
50. 24 h post seeding, fix cells with 4% PFA in PBS for 30 min at RT and wash with PBS twice.
51. Permeabilize cells with 0.2% Tween-20 (Sigma, P1379) in PBS for 5 min at RT and rinse with PBS before blocking with blocking buffer (3% BSA (Sigma, A7906)/PBS) for 1 h at RT.
52. Dilute primary antibody against CD9 in blocking buffer (1:200) and add to cells. Incubate cells with antibody overnight at 4°C.

***Note:*** Alternatively, CD63, ALIX, TSG101 and HSP90 may also be used as markers of multivesicular endosomes.^6^

53. The next day, rinse cells with PBS thrice and incubate with secondary antibody (dilution 1:500) and DAPI in blocking buffer for 30 min at RT.
54. After rinsing with PBS, mount the coverslips on microscope slides using Faramount mounting medium and investigate with confocal microscopy (Leica SP8, HC PL APO CS2 63×, NA 1.4, oil immersion, filter settings for DiI; excitation 551 nm, emission 565 nm and DAPI; excitation 358 nm, emission 463 nm, GFP; excitation 490 nm, emission 544 nm).

### Isolation of small extracellular vesicles from cell culture

**Timing: ∼ 3 days**

This section describes the isolation of small extracellular vesicles from donor cell conditioned media.

sEVs are isolated from the culture medium of recombinant cell lines expressing XP-GFP-DNAJB6b and XP-GFP.

**CRITICAL:** We recommend always making fresh sEV isolates for functional assays, including protein aggregation studies. You may store them overnight at 4°C, and use them the next day. However, do not keep them at 4°C for an extended period of time. We have observed that sEVs tend to aggregate over time. Storage of sEVs at -20°C is not recommended either because of the potential loss of functional activities.^7^

For nonfunctional assays such as sEV marker characterization with western blotting, you may freeze the samples in loading buffer and store at -80°C. Please refer to 57–69 for further details.

55. Seed 3–5 × 10^6^ C17.2 sEV donor cells i.e., high-expression monoclonal XP-GFP and XP-GFP-DNAJB6 cells per flask in 10 T162 flasks (Corning) in 20 mL complete medium at 37°C under 5% CO_2_ overnight.
56. The next day, wash away the old medium with HBSS and add 20 mL exosome-depleted medium to the cells (they should be about ∼40% confluent).
57. 48 h later, collect the medium (∼180 mL) in 5 UC tubes (∼36 mL/tube) and isolate extracellular vesicles by sequential centrifugation as follows.

58. Add an equal amount of sterile water in 1 UC tube which will be for balancing. Weigh the tubes to balance and proceed with centrifugation -
  a. Remove dead cells from the supernatant by centrifugation at 500 g for 10 min at 4°C.
    Carefully transfer the supernatant to a fresh falcon tube by pipetting without touching the pellet. The procedure can be performed at RT.
  b. Next, centrifuge the supernatant at 2,000 g for 10 min at 4°C to remove cellular debris (Beckman Coulter, Allegra X-15R). Carefully transfer supernatants into a UC tube (38.5 mL, Certified Free Open-Top Thinwall Polypropylene Tube, 25 × 89 mm) by pipetting without touching the pellet. The procedure can be performed at RT.
  c. Remove apoptotic vesicles and microvesicles by centrifugation at 10,000 g for 30 min at 4°C in Sorvall Discovery 90SE UC using Beckman SW32i rotor.
  d. Finally, subject the resultant supernatant to ultracentrifugation at 110,000 g for 70 min at 4°C to collect sEVs.
  e. Resuspend the pellet in 5 mL of cold DPBS, transfer the suspension into a new UC (14 mL, Certified Free Open-Top Thinwall Polypropylene Tube, 14 × 95 mm) and ultracentrifuge again by ultracentrifugation at the same conditions using Beckman SW55i rotor.
  f. Re-suspend the final pellet in 50 μL cold DPBS, and store the sEV suspension at 4°C for downstream processing.

### Characterization of small extracellular vesicles

**Timing: ∼ 2 days**

This section describes how to characterize the physicochemical properties of small extracellular vesicles using nanoparticle tracking analysis (NTA) and western blotting for assessing the enrichment of extracellular vesicle markers.

59. Measure the protein concentration of sEV suspension with DC protein assay kit I (Bio-Rad, 5000111; https://www.bio-rad.com/en-nl/sku/5000111-dc-protein-assay-kit-i?ID=5000111).
60. Perform NTA.
  a. Dilute 1 μg and 2 μg total extracellular protein samples in 1 mL of PBS.
  b. Use these solutions for measuring the size of particles by NanoSight LM14 using NTA software 3.0, Malvern, Worcestershire, United Kingdom following manufacturer’s protocol (https://www.malvernpanalytical.com/en/learn/knowledge-center/user-manuals/man0511en).
61. Western blot analysis – To ensure that the therapeutic proteins are present in the sEVs, prepare 30 μg whole cell lysates and sEV by mixing with 2× Laemmli loading buffer with SDS and protease inhibitors (Roche, 11697498001).
62. Boil the samples at 90°C for 5 min. There might be some condensation on the Eppendorf lid.
63. Spin down the tubes quickly and load the contents onto an SDS-PAGE gel. Run the gel at 200 V for 2 h.
64. Place the gel onto a PVDF membrane (Millipore, IPFL00010) in transfer buffer and allow for the transfer of the proteins at 500 mA for 1 h.
65. Block the membrane with Odyssey blocking buffer (Li-COR, 927-40000) for 1 h at RT.
66. For primary antibody incubation (Lamp2b, Calnexin, DNAJB6, and GFP), in a 10 cm square petri dish, place a parafilm with the size of the blot on the inner surface of the dish.

***Note:*** Parafilm, because of its hydrophobicity, will prevent spreading of the liquid and keep it close to the blot.

67. Add 300–500 μL of antibody solution (prepared in odyssey blocking buffer, 1:1000) on the parafilm.
68. Take out the blot from the blocking solution, cut a small piece off right corner to mark the protein side, and place the blot with the protein side facing the antibody solution i.e., facing downward.
69. Place one small wet tissue paper along the edges of the petri dish. Make sure that the tissue paper is not touching the blot, to prevent drying of the blot by capillary action.
70. Close the dish, and incubate the blots overnight at 4°C.
71. Next day, wash the blots with 0.1% PBS-Tween20 4 × 5 min.
72. Incubate the blots with secondary antibody solution (1:5000 in blocking buffer) for 1 h at RT, following the same procedure as for primary antibody incubation.
73. Wash with 0.1% PBS-Tween20 4 × 5 min.
74. Take out the blot from the petri dish and acquire images with an Odyssey® Infrared Imaging System (Li-COR) following manufacturer’s protocol (https://www.licor.com/bio/support/contents/applications/western-blots/fluorescent-western-blot-detection-protocol.html?Highlight=on-cell western assay).

### *In vitro* inclusion body assay (fluorescence microscopy)

**Timing: ∼ 4 days**

This protocol describes the procedure to make an *in vitro* HD cell model to assess the potency of DNAJB6-containing sEVs using fluorescence microscopy.

**CRITICAL:** As this is a functional assay, it is important that you use freshly isolated sEVs. We recommend seeding cells for the inclusion body assay on the day you prepare the western blot samples to check for the presence of DNAJB6 and EV markers. When their presence is confirmed, use the sEV isolates within 1–2 days.

***Note:*** Prewarm HBSS, trypsin, complete medium, and exosome-depleted medium.

75. Place the 13 mm glass coverslips in 24 well plate as described in 40–48.
76. Trypsinize Hek293T cells, count and seed 3 × 10^4^ cells in complete medium per well and incubate for 24 h at 37°C.
77. Transfect with 200 ng pEGFP-Q74 (encoding HTT-Q74-GFP) using JetPEI® (Polyplus,10110-N) for 4 h following the manufacturer’s protocol (https://www.polyplus-transfection.com/products/jetpei/?hilite=%27jetpei%27).
78. Rinse the cells rigorously with HBSS. HEK293T cells are easily detached from the well bottom; hence, wash multiple times but avoid cell loss.
79. Add 25 μg total sEV protein content to 500 μL of exosome-depleted medium (50 μg/mL per well) and add to the washed cells.
80. Incubate cells for 36 h. Take exosome-depleted medium without sEVs as control.

**CRITICAL:** It takes >8 h post-transfection for protein expression. As DNAJB6 acts upstream of aggregation i.e., it prevents aggregate formation but does not remove existing aggregates, it is important that you add sEV solution right after the jetPEI incubation for best results. For cargo proteins acting downstream of protein aggregation, sEVs can be added later after transfection (e.g., 24 h post-transfection).

81. Fix cells with 4% PFA (in PBS, pH 7.4) for 10 min at RT.
82. Rinse with PBS and add DAPI (1 μg/mL in PBS) for 20 min at RT directly followed by rinsing (PBS).
83. Mount the coverslips using Faramount mounting medium (Dako, The Netherlands, S302580).
84. Image samples using fluorescence microscopy. We used Leica DMI 6000B, HCX PL FLUOTAR L, 40× dry objective with numerical aperture 0.60. Set the excitation/emission settings to GFP (excitation 490 nm/emission 550 nm) and excitation 360 nm/emission 460 nm for DAPI.
85. For each sample, take at least 5 images from different areas randomly.

**Figure 5:**
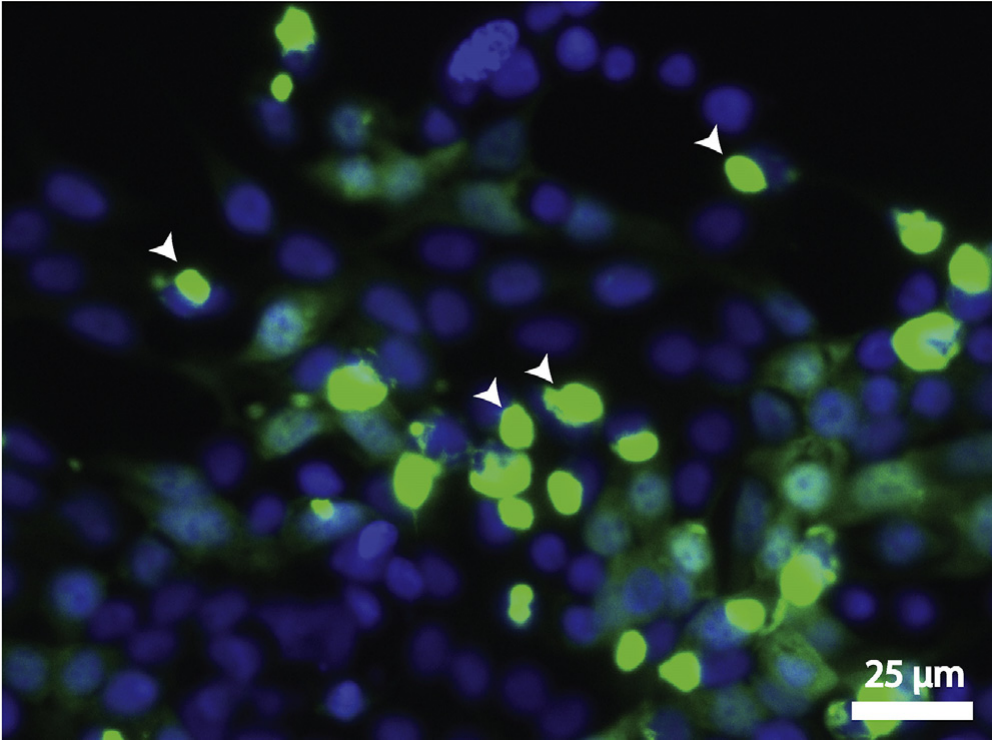
Representative image of HTT-Q74-GFP-expressing cells The bright green blobs represent inclusion bodies/aggregates, some of which are indicated by a white arrow.

***Note:*** As the inclusion bodies show very high intensity signal, it is advisable to start with low intensity of excitation. See Figure 5 for reference.

86. Analysis of inclusion body numbers –
  a. Count the number of nuclei using ImageJ Plugins > Analyze > Cell counter. Make sure that you only count cells that have been transfected.
  b. Count the number of inclusion bodies using the same plugin.
  c. Document all the numbers in an excel file.
  d. Get a ratio of the number of cells with inclusion bodies to the total number of transfected cells.
87. Repeat the experiment 3 times. Draw a bar graph using Graphpad PRISM or any other software for analsysis.

### Filter trap assay for analysis of protein aggregates

**Timing: ∼ 4 days**

**Figure 6:**
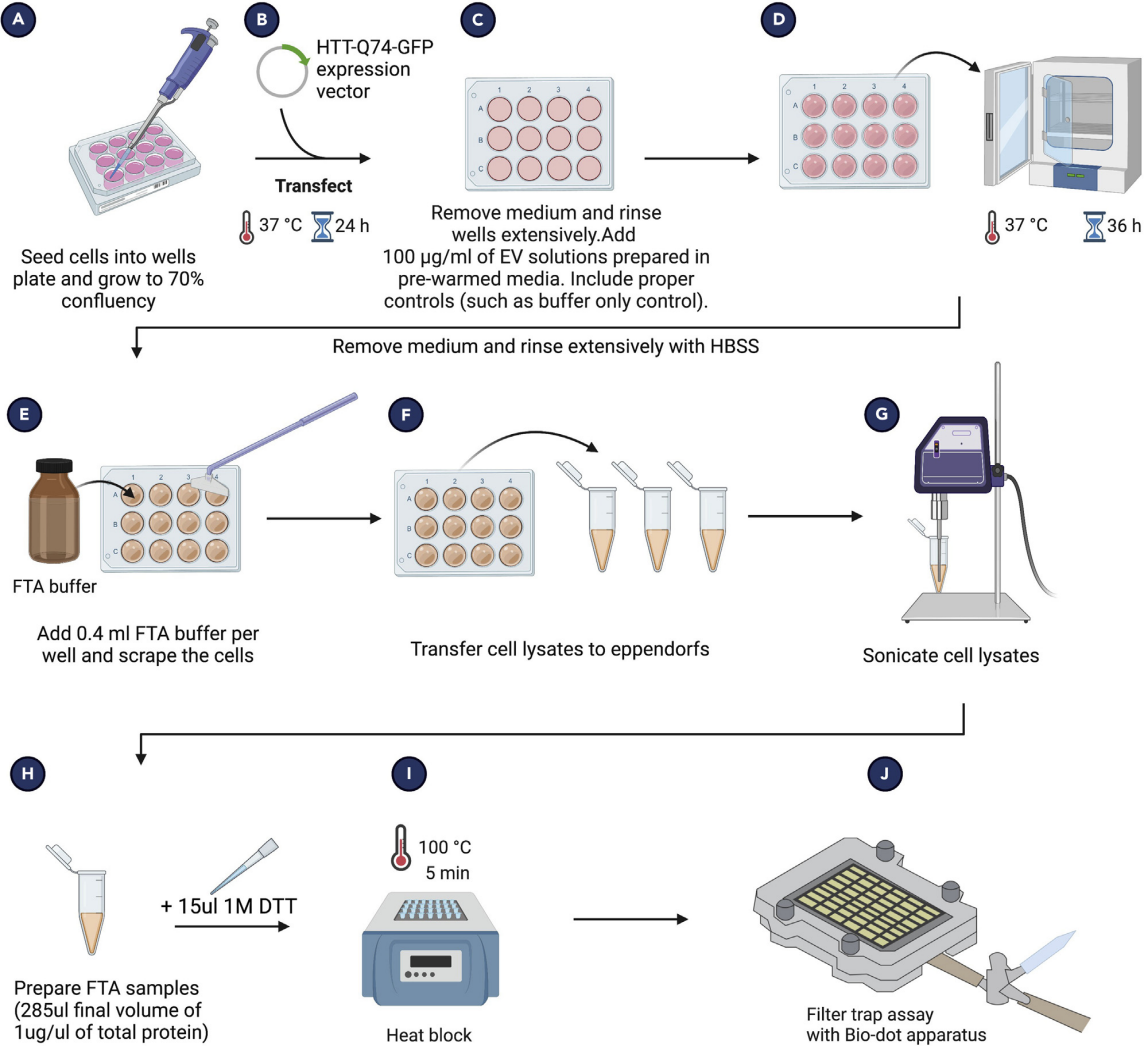
Schematic overview of the preparation of filter trap assay samples

**Figure 7:**
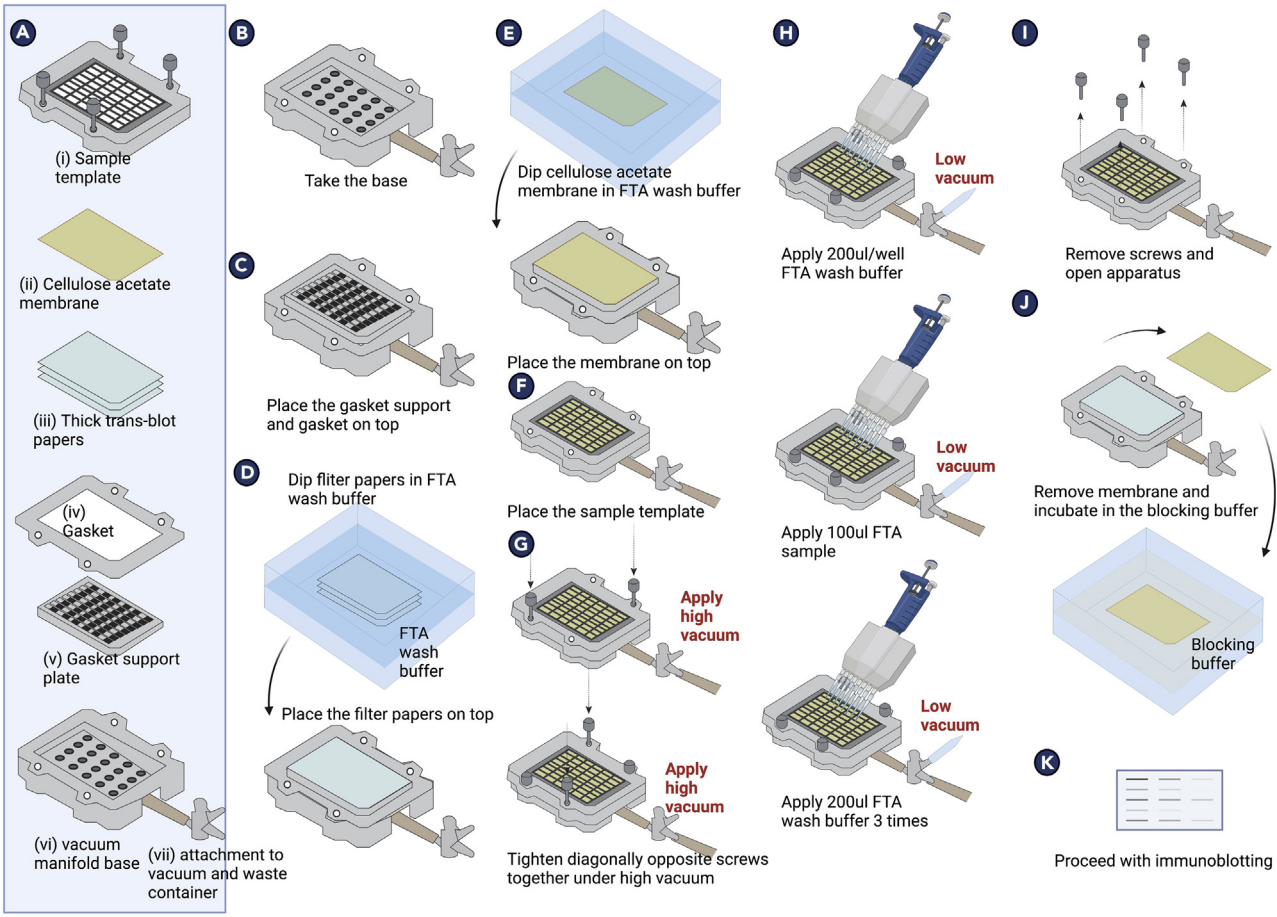
Schematic overview of filter trap assay procedure

This protocol explains the filter trap assay, which separates aggregated (insoluble) proteins from soluble proteins. Shortly, cell lysates (Figure 6) are passed through the cellulose acetate membrane which traps aggregates >200 nm, separating them from soluble proteins (Figure 7). The trapped proteins can be detected, which enables semiquantitative measurements for protein aggregation.

88. Place coverslips in 6 well plates as described in 40–48.
89. Trypsinize Hek293T cells, count and seed 15 × 10^4^ cells in complete medium per well and incubate for 24 h at 37°C.
90. Transfect with 1 μg pEGFP-Q74 (encoding HTT-Q74-GFP) using JetPEI® (Polyplus,10110-N) for 4 h following the manufacturer’s protocol.
91. Rinse the cells rigorously with HBSS. HEK293T cells are easily detached from the well bottom, hence wash multiple times but avoid cell loss.
92. Add 100 μg total sEV protein content to 1 mL of exosome-depleted medium (50 μg/mL conc. per well) and add to the washed cells. Incubate cells for 36 h. Take exosome depleted medium without sEVs as control.
93. After incubation, rinse cells with HBSS once.

**CRITICAL:** Because of the potential risk of transfer of protein aggregates to humans, utmost care should be taken while handling samples to prevent contact. Wear gloves at all times, and work in a fume hood from this point onwards.

94. Add 400 μL of filter trap assay (FTA) buffer with SDS (10 mM Tris-Cl pH 8.0, 150 mM NaCl, 2% SDS) per well.
95. Scrape the cells in each well. This will result in a very sticky mass. Transfer it to an Eppendorf carefully using 1 mL tips (200 μL tips do not transfer well enough).

***Note:*** Generally, cell lysates are prepared on ice. However, for filter trap assay samples, because of the presence of highly denaturing conditions such as 2% SDS, it is not required nor recommended to prepare samples on ice as this will lead to precipitation of SDS. Prepare samples at RT.

96. Sonicate the samples and measure protein concentration with DC protein assay kit (Bio-Rad, 5000111; https://www.bio-rad.com/en-nl/sku/5000111-dc-protein-assay-kit-i?ID=5000111).
97. Dilute the cell lysates in assay buffer to achieve 1 μg/μL of final concentration in a final volume of 285 μL.
98. Add 15 μL of 1 M DTT (MedChemExpress, HY-15917) to each sample to make the final total volume 300 μL.
99. Heat the samples at 95°C for 5 min.

***Note:*** Same samples are used for both filter trap assay and western blotting. Remaining sample can be stored at -80°C for later use.

100. Setting up of FTA Bio-Dot apparatus –
  a. Open the Bio-Dot SF apparatus (Biorad, 1706542). Make sure it is thoroughly cleaned with water and soap.
  b. Prepare FTA wash buffer (10 mM Tris-Cl pH 8.0, 150 mM NaCl, 0.1% SDS) and take ∼50 mL buffer in big plastic box.
  c. Soak 2 thick Trans-blot papers and place them on the base of the apparatus.
  d. Similarly, soak 0.2 μm cellulose acetate membrane in the same buffer and place it on the trans-blot papers.
  e. Remove all the air bubbles by rolling a 10 mL plastic pipette on the membrane by pressing gently.
  f. Add the lid of the apparatus and close the assembly by tightening the screws diagonally.
  g. Slowly apply full vacuum and tighten the screws further under vacuum.
  h. Switch off the pump, add 200 μL FTA wash buffer and apply vacuum until all buffer goes through.
  i. Repeat step once. Close the vacuum.
  j. Add 100 μL of the samples into each well.
  k. Apply low vacuum very carefully and let the buffer pass through completely.
  l. Wash the slots thrice as given in step h above.
  m. Make sure the buffer is completely gone.
101. In another big box, take 20 mL Odyssey Blocking buffer.
102. Open the apparatus, and remove the membrane, and place it in blocking buffer in a square petri dish for 1 h at RT on a shaker.
103. Rinse the blot with PBS-T and add anti-GFP antibody primary antibody solution (1:2000 in Odyssey blocking buffer) to the membrane. Incubate overnight at 4°C on a shaker.
104. The next day, wash the blot with PBS-T 4 × 10 min.
105. Prepare the Odyssey secondary antibody mixture (1:5000 in Odyssey blocking buffer).
106. Incubate the blot with the mix for 1 h at RT on a shaker.
107. Wash the blot with PBS-T 4 × 10 min.
108. Detect the proteins on the blot with Odyssey Infrared Imaging System.

### Western blot assay for the analysis of protein aggregation

**Timing: ∼ 2 days**

This section describes the functional assay of protein aggregation using western blotting.

in cells treated with extracellular vesicles loaded with DNAJB6.

***Note:*** It is important that you make these samples from freshly made isolates. However, once these are boiled with the loading buffer, they can be stored at -80°C until further use. However, avoid freeze-thaw cycles until use.

109. For western blotting, take 30 μg of total protein of each sample from step 99 and follow steps 61–74 using anti-GFP antibody with the following modifications.
  a. As the protein aggregates are too big to travel through running gel, they are trapped in the stacking gel. Hence, it is important to use the entire gel for transfer onto the PVDF membrane.
  b. The stacking gel easily breaks during transfer, hence lift and place the gel gently on the membrane. Rearrange the well walls into the original position if necessary.
110. Analysis of the western blot to quantify protein aggregation using ImageJ.
  a. Draw a rectangle around the bands in ImageJ in such a way that you can have a rectangle of the same size for all bands.
  b. Using Analyze > Measure, measure the band intensities of the protein aggregation in each sample.
  c. Calculate the extent of protein aggregation as a percentage with respect to control.

## Expected outcomes

The clinical translation of therapeutics for brain diseases, including Huntington’s disease, is hindered by the generally poor accumulation of therapeutics in the brain upon their systemic administration. Drug delivery vehicles are being developed to facilitate drug delivery at the target site. sEVs have emerged as promising biological delivery vehicles for various biomolecules, including proteins.^8^ In this protocol, we have described the procedure of loading sEVs with the potent anti-aggregation chaperone protein DNAJB6, and demonstrate their use in reducing aggregation in an *in vitro* cellular model for HD.

There are two main outcomes of this protocol –high loading of DNAJB6 in sEVs, and reduction in the amount of protein aggregation in polyQ expressing cells.

### High loading of DNAJB6 in sEVs

It is difficult to determine the exact amount of loaded DNAJB6 protein in each EV without performing further specialized biochemical experiments. If 10 μg of EVs can give a detectable signal of DNAJB6 in western blotting, the DNAJB6 expression levels would be sufficient to produce detectable decrease in protein aggregation in cells in the assays stated in this article. Hence, make sure to pick colonies that show maximum DNAJB6-GFP signal to maximize the probability of getting high loading in sEVs.

### Reduction in the amount of protein aggregation in polyQ expressing cells

In our hands the DNAJB6-loaded sEVs generated ∼30% decrease in protein aggregation in HD cellular model in both fluorescence microscopy and WB analyses.

## Limitations

The most problematic limitation, is the high volumes of conditioned media required to get sufficient amounts of sEVs for experiments. Although C17.2 cells produce large amounts of sEVs, bioreactors may be considered for higher and less labor-intensive production of sEVs.

In addition, due to (high) protein overexpression, overall cellular function may be impacted. As a consequence, it is possible that the EVs contain not only the protein of interest but also other components that can be either beneficial or non-beneficial to the process under study.

## Troubleshooting

### Problem 1

Low exogenous protein expression in stable cell lines (step 20).

### Potential solution

C17.2 cells are known to resist exogenous expression. But it is important to have high amounts of DNAJB6 in cells in order to maximize the sEV loading. An option is to use high(er) amounts of plasmid DNA during nucleofection. You may also increase the antibiotic concentration to 5 μg/mL in order to select only high DNAJB6 expressing cells.

### Problem 2

Low sEV yield (step 59).

### Potential solution

During sEV isolation procedure, the main loss takes place during washing of the sEV samples. One way to avoid this loss is to use less washing steps during ultracentrifugation or use smaller ultracentrifugation tubes. Another reason for a low sEV yield is the incomplete recovery of sEV pellets due to long handling times, especially when pellets are left in buffers for a longer period of time before resuspending. Hence, try to avoid long handling times, or leave the pellets in wash buffers instead of media supernatants to avoid loss during transfer.

### Problem 3

Variable sEV yield (step 59).

### Potential solution

We have often experienced variability in the sEV field. We have found that the major cause of this is variability in cell seeding and higher passage number. Use of different ultracentrifugation equipment within experiments also results in variability. Hence, try to attain similar and optimal cell density before harvest and use cells with passage number <40. Try to use the same equipment for all the isolates.

### Problem 4

Variable suppression levels in protein aggregation assay (step 108).

### Potential solution

Not often, but a few times, we have experienced some variability in the suppression levels of protein aggregation among different biological samples. The main cause of this is different quality of sEV samples. It is important to characterize the sEV isolate every time to confirm the levels of DNAJB6 and EV markers. If they attain the values similar to all other experiments, then only proceed to functional assays.

## Resource availability

### Lead contact

Requests for resources and reagents should be directed to the lead contact, Inge Zuhorn (i.zuhorn@umcg.nl).

### Materials availability

Plasmids and cell lines are available upon reasonable request.

### Data and code availability

This study did not generate/analyze datasets/code.
